# Supplementary material for: Psychological distress and problematic internet use among language teachers: a latent profile analysis
Source: PeerJ. 2025 Jul 23;13:e19707. doi: 10.7717/peerj.19707 (PMC12296581; doi:10.7717/peerj.19707)
Supplement: Supplemental Information 1 [file peerj-13-19707-s001.docx]

# Supplementary Material

**Table S1.** Step Summary of Multiple Logistic Regression.

| Model | | Action | Effect(s) | Model Fitting Criteria | Effect Selection Tests | | |
| --- | --- | --- | --- | --- | --- | --- | --- |
|  |  |  |  | -2 Log Likelihood | Chi-Square^a,b^ | df | Sig. |
| Step 0 | 0 | Entered | Intercept | 167.473 | . |  |  |
| Step 1 | 1 | Entered | Gender | 156.056 | 11.416 | 2 | .003 |
| Step 2 | 2 | Entered | Teaching experience | 148.814 | 7.242 | 2 | .027 |

Stepwise Method: Forward Stepwise

a. The chi-square for entry is based on the likelihood ratio test.

b. The chi-square for removal is based on the likelihood ratio test.
