# Supplementary material for: Psychological distress and problematic internet use among language teachers: a latent profile analysis
Source: PeerJ. 2025 Jul 23;13:e19707. doi: 10.7717/peerj.19707 (PMC12296581; doi:10.7717/peerj.19707)
Supplement: Supplemental Information 3 [file peerj-13-19707-s003.doc]

**Data Code**

| **Demography Section** | | | | | | | | | | | | | | | | |
| --- | --- | --- | --- | --- | --- | --- | --- | --- | --- | --- | --- | --- | --- | --- | --- | --- |
| gender | 1 = “male” | | | | | | | | 2 = “female” | | | | | | | |
| School type | 1 = “public school” | | | | | | | | 2 = “private school” | | | | | | | |
| School stage: | 1 = “junior high school” | | | | | | | | 2 = “primary school” | | | | | | | |
| Are you the homeroom teacher? | 1 = “yes” | | | | | | | | 2 = “no” | | | | | | | |
| Teaching experience: | 1 = “≤ 5 years” | | | | | | | | 2 = “>5 years” | | | | | | | |
| **DASS Section** | | | | | | | | | | | | | | | | |
| **Items** | **not at all** | | **some of the time** | | | **a good part of time** | | | | | | | **most of the time** | | | |
| S1 I found it hard to wind down | 0 | | 1 | | | 2 | | | | | | | 3 | | | |
| A1 I was aware of dryness of my mouth | 0 | | 1 | | | 2 | | | | | | | 3 | | | |
| D1 I couldn’t seem to experience any positive feeling at all | 0 | | 1 | | | 2 | | | | | | | 3 | | | |
| A2 I experienced breathing difficulty (e.g. excessively rapid breathing,breathlessness in the absence of physical exertion) | 0 | | 1 | | | 2 | | | | | | | 3 | | | |
| D2 I found it difficult to work up the initiative to do things | 0 | | 1 | | | 2 | | | | | | | 3 | | | |
| S2 I tended to over-react to situations | 0 | | 1 | | | 2 | | | | | | | 3 | | | |
| A3I experienced trembling (e.g. in the hands) | 0 | | 1 | | | 2 | | | | | | | 3 | | | |
| S3 I felt that I was using a lot of nervous energy | 0 | | 1 | | | 2 | | | | | | | 3 | | | |
| A4 I was worried about situations in which I might panic and make a fool of myself | 0 | | 1 | | | 2 | | | | | | | 3 | | | |
| D3 I felt that I had nothing to look forward to | 0 | | 1 | | | 2 | | | | | | | 3 | | | |
| S4 I found myself getting agitated | 0 | | 1 | | | 2 | | | | | | | 3 | | | |
| S5 I found it difficult to relax | 0 | | 1 | | | 2 | | | | | | | 3 | | | |
| D4 I felt down-hearted and blue | 0 | | 1 | | | 2 | | | | | | | 3 | | | |
| S6 I was intolerant of anything that kept me from getting on with what I was doing | 0 | | 1 | | | 2 | | | | | | | 3 | | | |
| A5 I felt I was close to panic | 0 | | 1 | | | 2 | | | | | | | 3 | | | |
| D5 I was unable to become enthusiastic about anything | 0 | | 1 | | | 2 | | | | | | | 3 | | | |
| D6 I felt I wasn’t worth much as a person | 0 | | 1 | | | 2 | | | | | | | 3 | | | |
| S7 I felt that I was rather touchy | 0 | | 1 | | | 2 | | | | | | | 3 | | | |
| A6 I was aware of the action of my heart in the absence of physical exertion (e.g. sense of heart rate increase, heart missing a beat) | 0 | | 1 | | | 2 | | | | | | | 3 | | | |
| A7 I felt scared without any good reason | 0 | | 1 | | | 2 | | | | | | | 3 | | | |
| D7 I felt that life was meaningless | 0 | | 1 | | | 2 | | | | | | | 3 | | | |
| **BSMAS Section** | | | | | | | | | | | | | | | | |
| **Items** | **very rarely** | | | **rarely** | | **sometimes** | | | | | **often** | | | | **very often** | |
| SD1 You spend a lot of time thinking about social media or planning how to use it. | 1 | | | 2 | | 3 | | | | | 4 | | | | 5 | |
| SD2 You feel an urge to use social media more and more. | 1 | | | 2 | | 3 | | | | | 4 | | | | 5 | |
| SD3 You use social media in order to forget about personal problems. | 1 | | | 2 | | 3 | | | | | 4 | | | | 5 | |
| SD4 You have tried to cut down on the use of social media without success. | 1 | | | 2 | | 3 | | | | | 4 | | | | 5 | |
| SD5 You become restless or troubled if you are prohibited from using social media. | 1 | | | 2 | | 3 | | | | | 4 | | | | 5 | |
| SD6 You use social media so much that it has had a negative impact on your job/studies. | 1 | | | 2 | | 3 | | | | | 4 | | | | 5 | |
| **SABAS Section** | | | | | | | | | | | | | | | | |
| **Items** | **strongly disagree** | **disagree** | | | **slightly disagree** | | | **slightly agree** | | | | **agree** | | | | **strongly agree** |
| PD1 My smartphone is the most important thing in my life. | 1 | 2 | | | 3 | | | 4 | | | | 5 | | | | 6 |
| PD2 Conflicts have arisen between me and my family (or friends) because of my smartphone use. | 1 | 2 | | | 3 | | | 4 | | | | 5 | | | | 6 |
| PD3 Preoccupying myself with my smartphone is a way of changing my mood (I get a buzz, or I can escape or get away, if I need to). | 1 | 2 | | | 3 | | | 4 | | | | 5 | | | | 6 |
| PD4 Over time, I fiddled around more and more with my smartphone. | 1 | 2 | | | 3 | | | 4 | | | | 5 | | | | 6 |
| PD5 If I cannot use or access my smartphone when I feel like, I feel sad, moody, or irritable. | 1 | 2 | | | 3 | | | 4 | | | | 5 | | | | 6 |
| PD6 If I try to cut the time I use my smartphone, I manage to do so for a while, but then I end up using it as much or more than before. | 1 | 2 | | | 3 | | | 4 | | | | 5 | | | | 6 |
| **IGDS9-SF Section** | | | | | | | | | | | | | | | | |
| **Items** | **never** | | | **rarely** | | | **sometimes** | | | **often** | | | | **very often** | | |
| GD1 Do you feel preoccupied with your gaming behavior? (Some examples: Do you think about previous gaming activity or anticipate the next gaming session? Do you think gaming has become the dominant activity in your daily life?) | 1 | | | 2 | | | 3 | | | 4 | | | | 5 | | |
| GD2 Do you feel more irritability, anxiety or even sadness when you try to either reduce or stop your gaming activity? | 1 | | | 2 | | | 3 | | | 4 | | | | 5 | | |
| GD3 Do you feel the need to spend increasing amount of time engaged gaming in order to achieve satisfaction or pleasure? | 1 | | | 2 | | | 3 | | | 4 | | | | 5 | | |
| GD4 Do you systematically fail when trying to control or cease your gaming activity? | 1 | | | 2 | | | 3 | | | 4 | | | | 5 | | |
| GD5 Have you lost interests in previous hobbies and other entertainment activities as a result of your engagement with the game? | 1 | | | 2 | | | 3 | | | 4 | | | | 5 | | |
| GD6 Have you continued your gaming activity despite knowing it was causing problems between you and other people? | 1 | | | 2 | | | 3 | | | 4 | | | | 5 | | |
| GD7 Have you deceived any of your family members, therapists or others because the amount of your gaming activity? | 1 | | | 2 | | | 3 | | | 4 | | | | 5 | | |
| GD8 Do you play in order to temporarily escape or relieve a negative mood (e.g., helplessness, guilt, anxiety)? | 1 | | | 2 | | | 3 | | | 4 | | | | 5 | | |
| GD9 Have you jeopardized or lost an important relationship, job or an educational or career opportunity because of your gaming activity? | 1 | | | 2 | | | 3 | | | 4 | | | | 5 | | |
